# Synergistic inhibition of human melanoma proliferation by combination treatment with B-Raf inhibitor BAY43-9006 and mTOR inhibitor Rapamycin

**DOI:** 10.1186/1479-5876-3-39

**Published:** 2005-10-28

**Authors:** Kerrington R Molhoek, David L Brautigan, Craig L Slingluff

**Affiliations:** 1 Department of Surgery, Division of Surgical Oncology, University of Virginia School of Medicine, Charlottesville, VA, USA; 2 Center for Cell Signaling, University of Virginia Health System, Charlottesville, VA, USA

**Keywords:** B-Raf, mTOR, melanoma, BAY43-9006, rapamycin

## Abstract

**Background:**

Targeted inhibition of protein kinases is now acknowledged as an effective approach for cancer therapy. However, targeted therapies probably have limited success because cancer cells have alternate pathways for survival and proliferation thereby avoiding inhibition. We tested the hypothesis that combination of targeted agents would be more effective than single agents in arresting melanoma cell proliferation.

**Methods:**

We evaluated whether BAY43-9006, an inhibitor of the B-Raf kinase, and rapamycin, an inhibitor of the mTOR kinase, would inhibit serum-stimulated proliferation of human melanoma cell lines, either alone or in combination. Proliferation was measured by quantitating melanoma cell numbers with a luciferase for ATP. Phosphorylation of proteins downstream of targeted kinase(s) was assayed by immunoblots. Statistical significance was determined with the Student-T test. Isobologram analysis was performed to distinguish additive versus synergistic effects of combinations of drugs.

**Results:**

Serum-stimulated proliferation of multiple human melanoma cell lines was inhibited by BAY43-9006 and by rapamycin. Melanoma cells containing the B-Raf mutation V599E were more sensitive than cells with wild-type B-raf to 10 nM doses of both BAY43-9006 and rapamycin. Regardless of B-Raf mutational status, the combination of low dose rapamycin and BAY43-9006 synergistically inhibited melanoma cell proliferation. As expected, rapamycin inhibited the phosphorylation of mTOR substrates, p70S6K and 4EBP1, and BAY43-9006 inhibited phosphorylation of ERK, which is dependent on B-Raf activity. We also observed unexpected rapamycin inhibition of the phosphorylation of ERK, as well as BAY43-9006 inhibition of the phosphorylation of mTOR substrates, p70S6K and 4EBP1.

**Conclusion:**

There was synergistic inhibition of melanoma cell proliferation by the combination of rapamycin and BAY 43-9006, and unexpected inhibition of two signaling pathways by agents thought to target only one of those pathways. These results indicate that combinations of inhibitors of mTOR and of the B-raf signaling pathways may be more effective as a treatment for melanoma than use of either agent alone.

## Background

In human cancers, mutant oncogenes are frequently associated with disease progression [1]. Thus, there is a need for development of effective therapies that can slow progression of solid tumors by blocking the action of those oncogenes. Cancer therapy has undergone a paradigm shift based on the therapeutic effectiveness of imatinib mesylate (Gleevec). This drug was designed as a specific inhibitor of the BCR-ABL oncogene protein tyrosine kinase, known to be responsible for chronic myeloid leukemia (CML) cells [2]. The therapeutic effectiveness of Gleevec and relative absence of detrimental side-effects has made it a model for the development of an array of new therapeutic agents targeted to inhibit signal transduction enzymes, especially protein kinases.

The recent discovery that 60–70% of human melanomas have activating mutations in B-Raf (with 80% of these mutations caused by a single substitution V599E) make this protein kinase an especially promising target for inhibition [3,4]. Indeed, lead compounds have been produced and tested, and currently are working their way through clinical trials. One example is BAY43-9006 (aka: sorafenib, *N*-(3-trifluoromethyl-4-chlorophenyl)-*N*'-(4-(2-methylcarbamoyl pyridin-4-yl)oxyphenyl)urea), an investigational compound, currently in phase II and III clinical trials, designed to inhibit both B-Raf and C-Raf kinases [5,6]. B-Raf is a component of a cell signaling pathway which includes the upstream activator of Raf, called Ras, and the direct substrate of Raf, called MEK1/2 and the MEK substrate ERK1/2 [7]. B-Raf phosphorylates MEK1 and MEK2 on Ser217 and Ser221, which activates it to dual phosphorylate ERKs, at Thr202/Tyr204 for human ERK1 and Thr185/Tyr187 for human ERK2 [8,9]. Mutations in RAF which cause constitutive activation of Raf kinase are thought to promote events leading to carcinogenesis. Pre-clinical and early phase I studies have suggested that BAY 43-9006 may be of therapeutic value not only in human tumors containing ras gene mutations, but also in tumors over-expressing growth factor receptors that activate the Ras/ERK pathway [10]. However, these studies have not addressed the effects of BAY 43-9006 in combination with any other kinase inhibitors.

Another molecular pathway commonly mutated (30–60%) in melanomas involves loss of the PTEN tumor suppressor gene, which can lead to constitutive activation of the mTOR kinase signaling pathway [11-13]. Inhibition of mTOR kinase is feasible with the macrolide natural product rapamycin (aka: sirolimus, RAPA, Rapamune, AY-22989, and NSC-226080). Rapamycin is an FDA-approved agent used as immunosuppressive therapy post organ transplant [14,15]. More recent clinical application of rapamycin has been with coated stents to suppress the neo-intima formation during restenosis in response to balloon angioplasty [16]. The action of rapamycin is understood to involve binding to the receptor protein FKBP12; this drug-protein complex binds to the mTOR protein kinase and interferes with phosphorylation of two well-recognized downstream targets, p70S6K (p70 ribosomal S6 kinase) and 4EBP1 (aka: 4E binding protein 1, eukaryotic translation initiation factor 4E binding protein, and PHAS-1) [17]. An appreciation of the potent inhibition of cell growth and protein synthesis, as well as cell cycle arrest, imposed by rapamycin led to testing of its derivatives, in particular CCI-779, as cytostatic agents, especially for various cancers refractory to other forms of cancer chemotherapy [18-20]. Pharmacokinetic analysis revealed that CCI-779 was progressively converted into rapamycin, its main metabolite, beginning as early as 15 minutes after infusion of the drug [20], therefore, we used rapamycin in our studies.

Our interest is in combining targeted agents for these pathways in an effort to determine if such treatments will be effective in the treatment of melanoma. We hypothesized that the combination of multiple targeted therapeutic agents would result in enhanced inhibition of melanoma cell proliferation compared to either drug alone, because of synergy between effects on two pathways. Here we show that serum-stimulated melanoma cell proliferation is inhibited by either rapamycin or BAY43-9006, with B-Raf V599E mutants showing an increased sensitivity to each drug at 10 nM compared to melanoma cells with wild-type B-Raf. Each of these drugs inhibited the serum-stimulated phosphorylation of known Raf and mTOR substrates. What was unexpected was that each of the drugs inhibited phosphorylation in both the Raf and mTOR pathways, suggesting there was interdependence or cross-talk between these pathways in melanoma cells. Furthermore, the combination of rapamycin with BAY43-9006 was synergistic compared to either drug alone at inhibiting proliferation of wild-type B-Raf and V599E mutant B-Raf melanoma cell lines.

## Methods

### Cell Culture

Melanoma cell lines used in this study were derived from tumors from patients at the University of Virginia (VMM5A, VMM18, and VMM39), as described previously [21,22]. All of the cell lines were cultured in RPMI 1640 medium supplemented with 5% fetal bovine serum, 2 mM L-glutamine, penicillin (100 units/ml), and streptomycin (100 μg/ml) at 37°C in 5% CO_2_, unless otherwise indicated. As a control, cells were incubated in Dulbecco's Phosphate buffered saline (PBS). VMM39 is a representative cell line from human melanomas known to contain a wild-type B-Raf gene and VMM18 and VMM5A both contain the V599E B-Raf activating mutation [23]. Other human melanoma cell lines listed in Table 1 include VMM12, a malignant melanoma cell line derived from tumors from a patient at the University of Virginia which is known to contain the V599E B-Raf activating mutation [23]. DM122 is a melanoma cell line derived from tumors from a patient at Duke University, and is known to contain a wild-type B-Raf gene (data not shown). DM6 and DM331 are melanoma cell lines derived from tumors obtained from patients at Duke University, however, their B-Raf status remains to be determined.

**Table 1:** Melanoma cell proliferation is stimulated by serum.

**Cell Line**	
VMM18	2.0
DM6	2.3
VMM39	2.1
VMM5A	2.0
VMM12	2.4
DM331	1.7
DM122	2.0

### Reagents and Inhibitors

The MEK1/2 inhibitor U0126 (Catalog # 662005) and BAY43-9006 (Catalog # 553011) were purchased from Calbiochem, and stock solutions were made in DMSO. Rapamycin (R-5000) was purchased from LC Laboratories and a stock solution was made in DMSO.

### Cell Proliferation Assays

**Figure 1:**
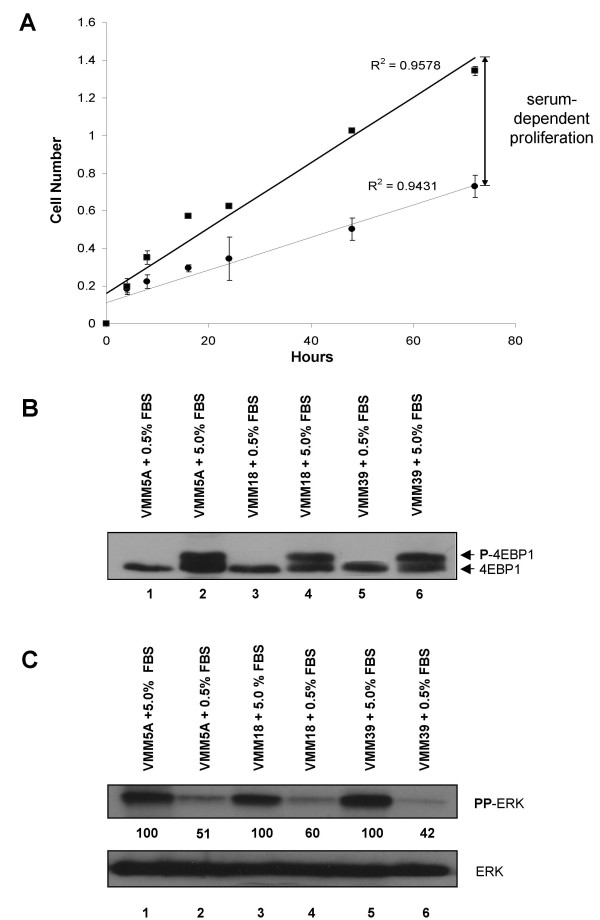
**A**, Growth curves of VMM18 melanoma cells in 5% and 0.5% serum-containing media. VMM18 melanoma cells were cultured in media containing either 5% or 0.5% serum and were assayed in triplicate at times 0, 4, 8, 16, 24, 48, and 72 hours using Cell Titer 96 Aqueous (Promega; Madison, WI) according to the directions supplied by the manufacturer. The absorbance at 490 nm (OD 490) measures the quantity of formazan product from the MTS-based assay and is directly proportional to the number of live cells present. The R^2 ^values for the linear regression lines determined using Microsoft Excel are listed above each line. The solid dark line with the squares represents the data collected from VMM18 melanoma cells grown in media with 5% serum. The thin line with the circles represents the data from VMM18 melanoma cells grown in media containing 0.5% serum. **B**, Western blot analysis of 4EBP1 from melanoma cell lines grown in 5.0% FBS and 0.5% FBS. Phosphorylation of 4EBP1 was assayed by its reduced migration in SDS-PAGE and the proteins were detected by immunoblotting. VMM5A, VMM18, and VMM39 cells were grown in media as indicated. **C**, Western blot analysis of ERK from melanoma cell lines grown in 5.0% FBS and 0.5% FBS. The dual phosphorylation of ERK was analyzed by phosophosite-specific immunoblotting in VMM5A, VMM18, and VMM39 melanoma cells cultured as described (upper panel). The total amount of ERK protein was determined by immunoblotting with a separate antibody (lower panel). The relative phosphorylation of ERK was quantitated by densitometry analysis using Image Quant 5.2 software and the values are given below the top panel.

Melanoma cells (25,000 cells per well) were plated in 96-well plates in RPMI plus either 5% FBS or 0.5% FBS, and cell numbers were assayed at time 0 and after 4, 8, 16, 24, 48, and 72 hours using Cell Titer 96 Aqueous (Promega Catalog# G3580; Madison, WI), according to the instructions provided by the manufacturer. Serum-dependent rates of growth were calculated using the slope of the lines from the growth curves, as shown in Figure 1A for VMM18. For experiments to examine the effects of the signal transduction inhibitors on serum-dependent melanoma cell proliferation, melanoma cells (1,000 cells per well) were plated in triplicate in a 96-well plates with 5% fetal bovine serum and allowed to adhere overnight. After 12–16 h, the cells were washed and treated with inhibitors as indicated for one hour. Cells were then washed and grown in RPMI medium with 5% FBS for 48 hours. Cell numbers were assayed with Cell Titer-Glo (Promega Catalog # G7571) according to the instructions provided by the manufacturer. The triplicate values were all within 5% and the mean values were calculated and plotted with error bars representing the standard deviation of triplicate samples from 3 independent experiments.

## Western Blot Analysis

**Figure 4:**
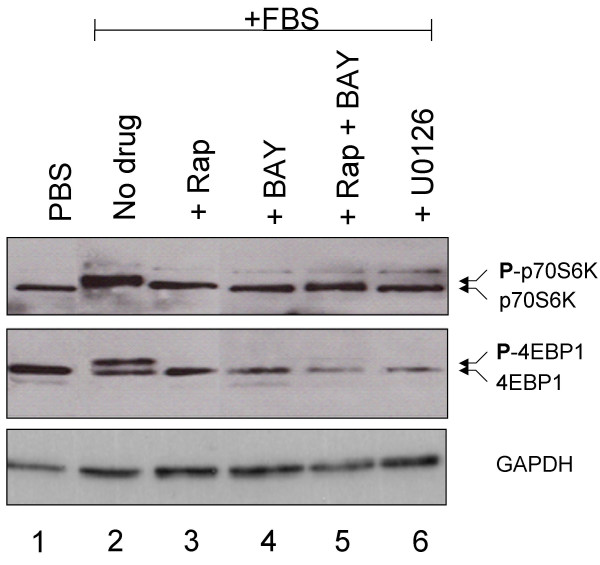
Phosphorylation of mTOR targets in VMM18 melanoma cells treated with rapamycin, BAY43-9006, or U0126. The phosphorylation of p70S6K and 4EBP1 was assayed by migration in SDS-PAGE and the proteins detected by immunoblotting. Control VMM18 cells grown in PBS without serum were analyzed in lane 1 and VMM18 melanoma cells in lanes 2–6 were grown 24 hours in media that contained 5% serum. Cells not treated with drugs (lanes 1 and 2) were compared to cells pre-treated for one hour with rapamycin (lane 3), BAY43-9006 (lane 4), a combination of rapamycin and BAY43-9006 (lane 5), or U0126 (lane 6). GAPDH was immunoblotted as a loading control (bottom panel).

**Figure 5:**
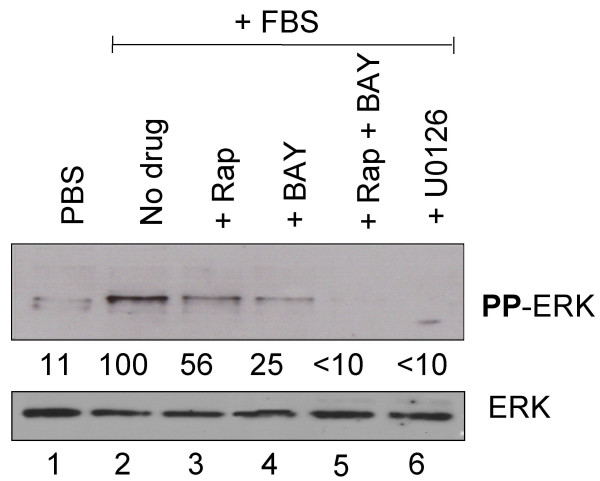
Phosphorylation of ERK in VMM18 melanoma cells treated with rapamycin, BAY43-9006, or U0126. The phosphorylation of ERK was analyzed by phosophosite-specific immunoblotting in VMM18 melanoma cells cultured and treated as described in Figure 4. The total amount of ERK protein was determined by immunoblotting with a separate antibody. The relative phosphorylation of ERK was quantitated by densitometry analysis using Image Quant 5.2 software and the values are given below the top panel.

For analysis of proteins in Figure 1B and 1C, VMM5A, VMM18, and VMM39 melanoma cells were plated in Petri dishes and incubated for 24 hours in either RPMI medium plus 5% FBS or 0.5% FBS. After 24 hours, the cells were harvested and lysed as described for analysis of proteins in Figures 4 and 5. For analysis of the proteins in Figures 4 and 5, VMM18 melanoma cells were plated in petri dishes, treated with drugs or not for one hour, washed, and incubated overnight in RPMI medium plus 5% FBS. The next day, cells were rinsed with room temperature PBS, frozen by placing the dish on a mixture of acetone and dry ice. Cells were lysed in one ml of ice-cold 5% trichloroacetic acid for 10 minutes, scraped from the dish using a Costar cell lifter and the slurry was transferred to a 1.5-ml microcentrifuge tube and centrifuged for 10 minutes at 10,000 × *g*. The supernatant was discarded, and the pellet was washed twice with cold acetone to extract away the trichloroacetic acid and the proteins resuspended in resolubilization buffer (20 mM Tris, 23 mM glycine, 1 mM EDTA, and 10 mM β-glycerophosphate). Protein yields were determined by BCA analysis. Proteins were resuspended in SDS-containing sample buffer, heated for 10 min at 100°C, and 10 ng/lane was resolved by SDS-PAGE and transferred to Immobilon-P (Millipore). Membranes were blocked in 1% BSA in 50 mM Tris-Cl (pH 7.5), 0.9% NaCl, 0.05% Tween 20, and 0.01% antifoam A. Membranes were probed with antibodies listed below. Proteins were detected with Pierce SuperSignal West Pico Chemiluminescent substrate (#34080) as recommended by the manufacturer and used to expose to Kodak BioMax film. Films exposed within the linear response range were scanned and used for densitometry analysis by Image Quant 5.2.

### Antibodies

Anti-p70S6 Kinase, clone SB20 Antibody (Catalog # 05-781, used at 1:8000) was purchased from Upstate. 4E-BP1 Antibody (Catalog #9452, used at 1:500) was purchased from Cell Signaling. GAPDH Antibody (Catalog # MAB374, used at 1:500) was purchased from Chemicon International. Anti-phospho MAP Kinase (ERK1/2), clone 12D4 antibody (Catalog # 05-481, used at 1:500) was purchased from Upstate. Anti-MAP Kinase 2/ERK2 antibody (Catalog #06-333, used at 1:500) was also purchased from Upstate. Phospho-MEK1/2 (Ser217/221) Antibody (Catalog#2354S, used at 1:1000) was purchased from Cell Signaling. Anti-Mouse IgG, peroxidase-linked species-specific whole antibody from sheep, secondary antibody (Catalog #NA931, used at 1:5000) was purchased from Amersham Biosciences. Anti-rabbit IgG, peroxidase-linked species-specific whole secondary antibody from donkey (Catalog # NA934, used at 1:5000) was also purchased from Amersham Biosciences.

### Isobologram Analysis

To assess whether a combination dose of rapamycin and BAY43-9006 is synergistic or simply additive, a focused isobologram method was used as described previously [24]. An IC70 was selected, and these doses of each drug alone were plotted as the ordinate and abscissa in a Cartesian log-log plot. The straight line connecting these IC70 values is the locus of points (dose pairs) that produce a simply additive combination. In an isobologram, the IC70 dose pairs for two drugs together which fall on the line indicate an additive effect. Points above this line indicate antagonism, and points below the line indicate synergism.

### Human Subjects

All of the research involving human subjects was approved by the University of Virginia's IRB (Human Investigation Committee) in accordance with assurances filed with and approved by the Department of Health and Human Services. Informed consent was obtained from all of the study participants.

## Results

### Proliferation of melanoma cells expressing wild-type and V599E mutant B-Raf

We examined the serum-dependent proliferation of various human melanoma cell lines. Figure 1 A is a growth curve for the VMM18 cell line, which is representative of the growth curves generated for each of the cell lines from a collection of human melanomas (Table 1). Cell proliferation was determined by the number of cells at 0, 4, 8, 16, 24, 48, and 72 hours, quantitated using the Cell Titer 96 Aqueous (Promega Corporation, Madison, WI) assay which measures reduction of MTS (a novel tetrazolium compound). These human melanoma cell lines proliferated even in limiting serum (0.5%). However, all showed higher rates of proliferation ~ 2-fold in the presence of 5% serum.

We could detect activation of the mTOR and ERK signaling pathways in proliferating melanoma cells. Shown in Figure 1 B is a Western blot detecting the phosphorylation of the mTOR substrate, 4EBP1, from 3 different melanoma cell lines grown in the presence of either 5% or 0.5% serum. The phosphorylation of 4EBP1 has previously been demonstrated to retard migration in SDS-PAGE [25], seen as the upper band in the doublet in the even numbered lanes (Figure 1B). Shown in Figure 1 C is a Western blot detecting both the dual phosphorylated (activated) form of ERK, as well as total ERK protein in three different melanoma cell lines grown in 5% or 0.5% serum. The quantitation of the relative phosphorylation of ERK relative to total ERK is shown between the blots, demonstrating about a two-fold increase in phosphorylation. The phosphorylation of ERK paralleled the relative increase in proliferation for each of these cell lines.

### BAY43-9006 and rapamycin inhibit proliferation of melanoma cells

**Figure 2:**
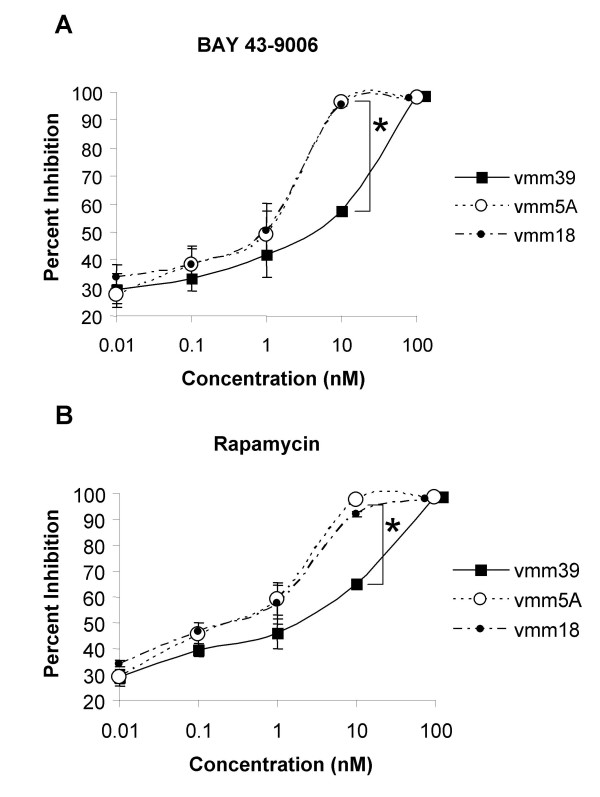
Inhibition of various melanoma cell lines by BAY43-9006 and rapamycin. **A**, The serum-stimulated proliferation of VMM18, VMM5A, and VMM39 melanoma cells was assayed in triplicate by Cell Titer Glo (Promega; Madison, WI). Inhibition was determined as the difference between the number of cells in the control without any added drug compared to the number of cells following treatment with an inhibitor. Cells were treated for one hour with doses of BAY43-9006 ranging from 0.01 nM to 100 nM and were assayed for serum-stimulated proliferation after 48 hours. The mean value for triplicate samples from three independent experiments is plotted relative to the control (treated with vehicle, DMSO). Error bars are present at each point on the graph. The Student T test was performed and the bracket and asterisk represents a statistically significant difference between VMM39 (wt) and VMM5A (V599E) and VMM18 (V599E) cells at the 10 nM concentration with a p value < 0.002. **B**, The serum-stimulated proliferation of VMM18, VMM5A, and VMM39 melanoma cells were assayed as described above except melanoma cells were treated with rapamycin instead of BAY43-9006 with doses ranging from 0.01 nM to 100 nM. The mean value for triplicate samples from three independent experiments is plotted. Error bars are present at each point on the graph. The Student T test was performed and the bracket and asterisk represents a statistically significant difference between VMM39 (wt) and VMM5A (V599E) and VMM18 (V599E) cells at the 10 nM concentration with a p value < 0.002.

We examined the serum-dependent proliferation of multiple human melanoma cell lines and the effects of inhibition of B-Raf by BAY43-9006 and of mTOR by rapamycin. Melanoma cell lines were tested for proliferation after treatment with a single dose of BAY43-9006 or rapamycin using Cell Titer-Glo (Promega Corporation, Madison, WI), a luminescence-based ATP cell viability assay. Cells were exposed to different doses of either drug for one hour. Then, the media was changed and the cells were cultured for two days in the presence of serum. We found that micromolar concentrations were cytotoxic, because cell numbers decreased after two days, whereas nanomolar concentrations were growth inhibitory. Melanoma cells showed dose-dependent inhibition with 0.01 nM to 100 nM of BAY43-9006 (Figure 2A), or rapamycin (Figure 2B). Proliferation of the cells was inhibited in either 5% or 0.5% serum.

Among the melanoma cell lines, there was a significant difference in the amount of inhibition at 10 nM BAY43-9006 or rapamycin. We observed that melanoma cell lines that contain the V599E mutation in B-Raf (VMM5A and VMM18) were more sensitive to BAY43-9006 and to rapamycin, compared to cell lines with wild-type B-Raf (VMM39). This difference in growth inhibition was observed in two additional cell lines, one wild-type (DM122) and one V599E (VMM12). Therefore, nanomolar concentrations of either BAY43-9006 or rapamycin inhibit the proliferation of melanoma cells, whether or not they have mutated B-Raf.

### Combining Rapamycin with BAY43-9006 synergistically inhibits serum-dependent proliferation of melanoma cells

**Figure 3:**
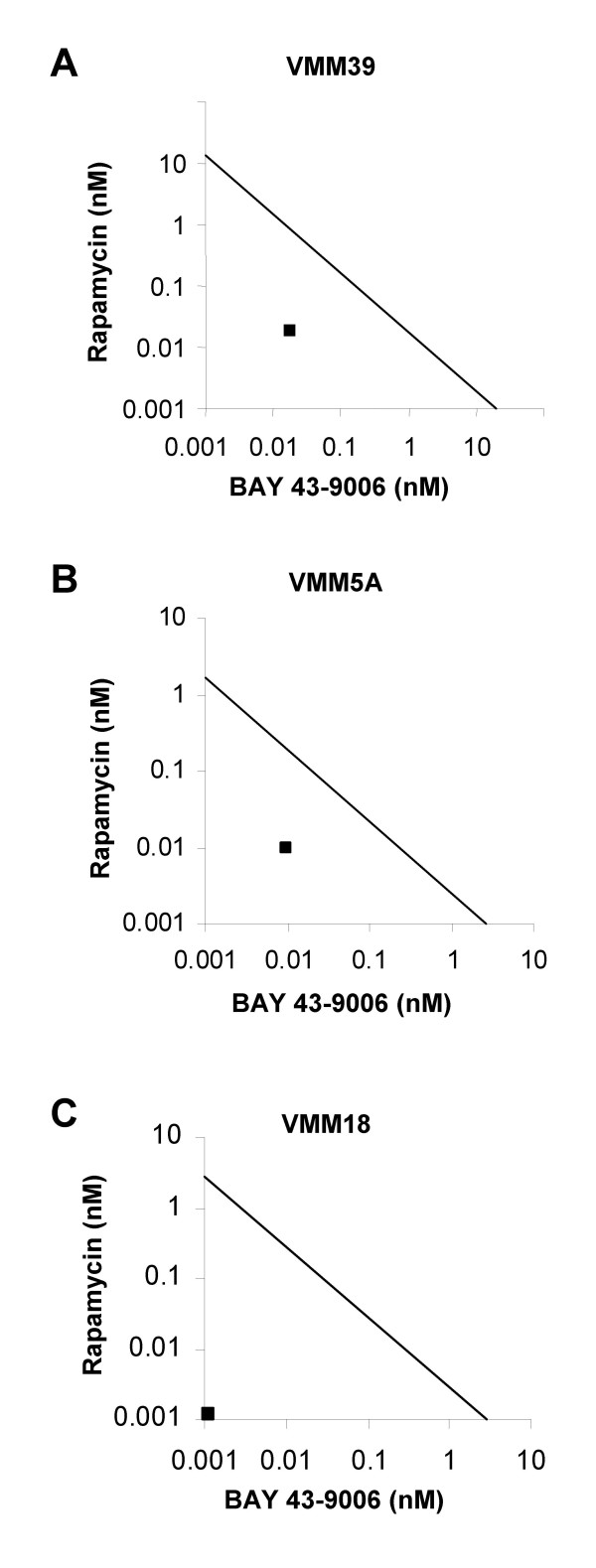
Isobologram analyses of inhibition of melanoma cell proliferation. **A**, VMM39 melanoma cells, **B**, VMM5A melanoma cells, and **C**, VMM18 melanoma cells. The straight line connecting the IC70 points (additivity line) is the locus of all dose pairs that should give the same effect. A dose pair (data point) below the line is synergistic, a dose pair on the line is additive, and a dose pair above the line is antagonistic. Note: all graphs are on a log-scale.

Melanoma cell proliferation was inhibited by either BAY43-9006 or rapamycin over the 0.01 – 100 nM concentration range. A combination of the two drugs was markedly more effective than either drug alone at inhibiting serum-stimulated melanoma cell proliferation. For example, 0.01 nM of each drug together was more effective at inhibiting melanoma cell proliferation than 1 nM of either drug alone. To assess synergism versus additivity quantitatively, we used a focused isobologram method (Figure 3). Treatment of three melanoma cell lines (VMM39, VMM5A, and VMM18) with rapamycin alone induced a 70% growth inhibition (IC70) from approximately 10 nM (VMM39) to 2 nM (VMM5A and VMM18). These were plotted on the ordinate. The IC70 concentration for BAY43-9006 alone was in the range of approximately 5 to 10 nM, in different cell lines, and these were plotted on the abscissa. Compared to the single agents, the IC70 for the dose pairs (rapamycin and BAY43-9006 together) falls below the line, for each of these melanoma cell lines, indicating that the combination is synergistic (Figure 3). Furthermore, VMM18, which contains the V599E substitution, was more sensitive to the combination treatment than melanoma cell lines with wild-type B-Raf, consistent with the enhanced sensitivity at the 10 nM dose of each agent (Figure 2 above). However, all melanoma cell lines tested displayed synergistic inhibition of proliferation, indicating that these drugs were more effective in combination than alone.

### Rapamycin and BAY43-9006 inhibit phosphorylation of proteins in the mTOR signaling pathway in melanoma cells

Melanoma cells were treated with rapamycin and BAY 43-9006, either singly or in combination, for one hour, and protein phosphorylation was examined by Western blot analysis 24 hours later. Rapamycin is an inhibitor of mTOR kinase and reduces phosphorylation of its substrates, p70S6K and 4EBP1 [17]. BAY 43-9006 is a chemical inhibitor of B-Raf kinase and reduces phosphorylation of MEK and ERK [26,27]. VMM18 melanoma cells grown in the presence of 5% serum had enhanced phosphorylation of p70S6K and 4EBP1 (Figure 4, lane 2) relative to cells grown in the absence of serum (Figure 4, PBS, lane 1). The phosphorylation of p70S6K and 4EBP1 retards migration in SDS-PAGE. Antibodies to these proteins were used to show all the protein and therefore enable evaluation of the fraction phosphorylated under different conditions. Treatment of VMM18 melanoma cells with a 10 nM dose of rapamycin inhibited the serum-stimulated phosphorylation of p70S6K and 4EBP1 (Figure 4, lane 3). Parallel treatment of VMM18 melanoma cells with a 10 nM dose of BAY43-9006 unexpectedly inhibited serum-stimulated phosphorylation of p70S6K and 4EBP1 (Figure 4, lane 4). There is not a well-documented requirement of Raf-MEK-ERK activity for the phosphorylation of mTOR substrates p70S6K and 4EBP1. Combination treatment with a 10 nM dose of rapamycin plus a 10 nM dose of BAY43-9006 blocked phosphorylation of p70S6K and 4EBP1 as effectively as either drug alone (Figure 4, lane 5). Thus, even though cell proliferation was suppressed more effectively by this combination of drugs, this was not reflected in a detectable further decrease in phosphorylation of the mTOR target proteins p70S6K and 4EBP1. As an additional control, we treated VMM18 melanoma cells with U0126, a MEK inhibitor, which blocked serum-stimulated phosphorylation of both p70S6K and 4EBP1 (Figure 4, lane 6). This result showed that MEK/ERK activities contribute to phosphorylation of p70S6K and 4EBP1.

We noted that total 4EBP1 in cells treated with a combination of rapamycin plus BAY43-9006, or with U0126, was lower relative to untreated cells or cells treated with either rapamycin or BAY43-9006 alone. Equal recovery of other proteins from the cells was demonstrated by immunoblotting both for p70S6K and for GAPDH, used as a loading control. We do not understand the basis for the reduced recovery of 4EBP1, but it did not seem to depend simply on the phosphorylation state because phosphorylation was blocked with the single drug treatments, without change in the level of the 4EBP1 protein.

### Rapamycin and BAY43-9006 inhibit phosphorylation of proteins in the B-Raf-MEK-ERK signaling pathway in melanoma cells

In VMM18 melanoma cells, the dual phosphorylation (Tyr/Thr) of ERK was 9-fold higher in cells grown in 5% serum relative to cells grown in the absence of serum (Figure 5, lanes 2 versus 1). There also was an increased level in the dual phosphorylation (Ser217/221) of MEK (not shown). Treatment of VMM18 melanoma cells with a 10 nM dose of BAY 43-9006 produced a 75% decrease in the dual phosphorylation of ERK (Figure 5, lane 4) and reduced the phosphorylation of MEK below detection levels (not shown). These results were consistent with the inhibition of B-Raf by BAY43-9006. On the other hand, when VMM18 melanoma cells were treated with a 10 nM dose of rapamycin, the dual phosphorylation of ERK was reduced by about half (Figure 5, lane 3). Our interpretation of this result is that mTOR activity is required to maintain the phosphorylation of ERK in melanoma cells. Combination treatment of a 10 nM dose of rapamycin plus a 10 nM dose of BAY 43-9006 reduced the phosphorylation of ERK to a level even below that seen in cells grown in the absence of serum (Figure 5, lane 5). This inhibition of ERK phosphorylation by combination of rapamycin and BAY43-9006 was as effective as inhibition of MEK by the U0126 compound (Figure 5, compare lanes 5 and 6).

## Discussion

New cancer treatments involve directly targeting enzymes essential for the growth and proliferation of cancer cells. The mTOR pathway regulates cell growth, and the Raf/MEK/ERK pathway is critical for cell proliferation. Activating mutations in B-Raf have been found in 60–70% of human melanomas, making B-Raf a potential target for small molecule inhibitors as therapy [3,4,11,28]. Indeed, new drugs such as BAY43-9006 have been developed as selective inhibitors of B-Raf and are currently in Phase II clinical trials [6]. Inhibition of mTOR by rapamycin has been standard treatment for immunosuppression following organ transplant [15], and the rapamycin derivative CCI-779 is now being clinically tested as a cancer chemotherapy [18-20]. Thus, B-Raf and mTOR are acknowledged targets for anti-proliferative therapy [29].

Current knowledge suggests that B-Raf and mTOR protein kinases operate in separate signaling pathways. The B-Raf kinase is activated by GTP-Ras in response to growth factors and phosphorylates MEK, which in turn activates ERK to phosphorylate downstream targets such as kinases and transcription factors that promote cell division [30]. The mTOR kinase responds to both nutrient and growth factor signals to activate p70S6K and 4EBP1 to increase protein translation as part of a cell growth response [31]. Increase in cell growth (size) is a pre-requisite for cell proliferation. Because the B-Raf and mTOR pathways are thought to operate in parallel, we hypothesized that combined inhibition of these kinases would be effective in blocking cell growth and cell proliferation. Though our results with multiple melanoma cell lines support that hypothesis, they also gave some unexpected results.

Human tumors deficient in PTEN have activated Akt, and are especially sensitive to mTOR inhibitors [13]. However, pharmacogenomic profiling indicates that melanomas are not, in general, PTEN deficient and therefore would be unresponsive to mTOR inhibitors. Results from a phase II trial using CCI-779 alone showed only one response among 33 observed patients [32]. These data suggest that CCI-779 is not sufficiently active in melanoma as a single agent. However, our data show that melanoma cell proliferation is effectively inhibited in vitro by low doses of rapamycin. Together, these findings argue against use of CCI-779 as a single agent, but support investigation of mTOR inhibitors as part of combination therapy for treatment of patients with malignant melanoma.

With regards to B-Raf, recent structural studies have shown that BAY43-9006 interacts with an inactive conformation of B-Raf [33]. In biochemical assays, the kinase activity of V599E B-Raf is less sensitive to inhibition by BAY43-9006 than wild-type B-Raf, suggesting that melanomas with the B-Raf V599E mutation might be resistant to the effects of this drug [33]. However, in the present study, proliferation of the human melanoma cells was inhibited by BAY43-9006, and at a dose of 10 nM, the cells that contained mutated B-Raf V599E were more sensitive than cells with wild-type B-Raf. In clinical studies with BAY43-9006 plus chemotherapy, objective tumor regressions were more common in patients who had wild-type B-raf [34]. The findings of the current report support continued investigation of BAY43-9006 for treatment of patients with melanoma, and suggest that clinical effects observed may be due to some effects that are independent of B-raf kinase activity.

We found that multiple human melanoma cell lines proliferated in culture at different relative rates in the absence of serum and that the addition of serum to the medium doubled the rate of proliferation. Thus, we could use the consistent serum response to compare cell growth and proliferation with a variety of melanoma cell lines. At concentrations in the nanomolar range, we observed dose-dependent inhibition of cell proliferation by either rapamycin or BAY43-9006. In every cell line examined, combination of BAY43-9006 and rapamycin produced synergistic inhibition of cell proliferation compared to either drug alone. This suggests that administration of a combination of an mTOR inhibitor (rapamycin or CCI-779) and BAY43-9006 could be an especially effective approach to therapy of melanoma.

Our results indicate that rapamycin and BAY43-9006 inhibit their cognate targets in melanoma cells (mTOR and B-Raf respectively), as well as downstream effectors thought to be in other pathways, providing evidence for cellular cross-talk between the different signaling pathways studied. Specifically, we found that BAY43-9006 inhibited serum-stimulated phosphorylation of p70S6K and 4EBP1, and rapamycin blocked serum-stimulated phosphorylation of ERK. Previously published results have suggested interdependence between mTOR and Raf-MEK-ERK signaling [35-40]. In vascular smooth muscle cells under hyperglycemic conditions (25 mM versus 5 mM glucose), inhibition of PI3K with LY294002 or inhibition of mTOR by rapamycin reduced the level of ERK Tyr-phosphorylation [35]. In cardiomyocytes, PKC-dependent activation of mTOR and p70S6K was inhibited by U0126, implicating a requirement for MEK [36]. Rapamycin inhibited the FGF-2 induced proliferation of two different small cell lung cancer lines (SCLC), whereas PD098059 inhibited one and not the other [37]. Combination of rapamycin and PD098059 was not tested. In proximal tubular epithelial cells, insulin-activated phosphorylation of 4EBP1 could be inhibited by PD098059, suggesting a requirement for MAPK [38]. Another report shows that following hypertonic stress, HEK 293 cells show increase in protein synthesis, and simultaneous inhibition of both mTOR and ERK was required to prevent *de novo *translation [39].

Since there appears to be cross-talk between mTOR and Raf-MEK-ERK pathways, it might be expected that combination therapy with rapamycin and BAY43-9006 might simply be additive. To our knowledge, the effects of combining inhibitors of these two pathways on proliferation of melanoma cells had not previously been examined. However, studies are in development for such combination therapies in human clinical trials, sponsored by the Clinical Trials Evaluation Program (CTEP) of the NIH. In the present study, we found that the combination of inhibitors synergistic for inhibition of melanoma cell proliferation.

Cancer cells may be dependent on particular oncogenes for cell growth, which renders them sensitive to drugs that inhibit these protein targets. Under these circumstances, single chemical inhibitors are efficacious, such as Gleevec inhibition of BCR-ABL in CML [2]. However, in a number of different cancers, single drug targeted therapy is only effective in about half of the patients [41]. These cancer cells utilize either alternate pathways or compensatory mechanisms to evade inhibition. Under these circumstances, combination therapy that inhibits different pathways may be especially effective. Our results show synergistic inhibition of cell proliferation with drugs against different pathways. Further, we exposed effects on pathways not thought to be targeted by agents currently used in the clinic. Because a combination of rapamycin and BAY43-9006 is more effective at inhibiting melanoma cell proliferation than either drug alone, further studies of this combination in animal models and clinical trials deserve to be examined.

## Competing interests

The author(s) declare that they have no competing interests.
